# Ca^2+^ entry via TRPC1 is essential for cellular differentiation and modulates secretion via the SNARE complex

**DOI:** 10.1242/jcs.231878

**Published:** 2019-07-01

**Authors:** Anne Schaar, Yuyang Sun, Pramod Sukumaran, Thad A. Rosenberger, Danielle Krout, James N. Roemmich, Lutz Brinbaumer, Kate Claycombe-Larson, Brij B. Singh

**Affiliations:** 1 Department of Biomedical Science, School of Medicine and Health Sciences, University of North Dakota, Grand Forks, ND 58203, USA; 2 US Department of Agriculture-Agricultural Research Service, Grand Forks Human Nutrition Research Center, Grand Forks, ND 58203, USA; 3 Neurobiology Laboratory, NIHES, NIH, 111 T.W. Alexander Drive, Research Triangle Park, NC 27709, USA; 4 Institute of Biomedical Research, (BIOMED) Catholic University of Argentina, Av. Alicia Moreau de Justo 1300, Edificio San Jose Piso 3, Buenos Aires C1107AAZ, Argentina

**Keywords:** TRPC1, Ca^2+^ entry, Adipocyte differentiation, Adiponectin secretion, Metabolic homeostasis

## Abstract

Properties of adipocytes, including differentiation and adipokine secretion, are crucial factors in obesity-associated metabolic syndrome. Here, we provide evidence that Ca^2+^ influx in primary adipocytes, especially upon Ca^2+^ store depletion, plays an important role in adipocyte differentiation, functionality and subsequently metabolic regulation. The endogenous Ca^2+^ entry channel in both subcutaneous and visceral adipocytes was found to be dependent on TRPC1–STIM1, and blocking Ca^2+^ entry with SKF96365 or using TRPC1^−/−^ knockdown adipocytes inhibited adipocyte differentiation. Additionally, TRPC1^−/−^ mice have decreased organ weight, but increased adipose deposition and reduced serum adiponectin and leptin concentrations, without affecting total adipokine expression. Mechanistically, TRPC1-mediated Ca^2+^ entry regulated SNARE complex formation, and agonist-mediated secretion of adipokine-loaded vesicles was inhibited in TRPC1^−/−^ adipose. These results suggest an unequivocal role of TRPC1 in adipocyte differentiation and adiponectin secretion, and that loss of TRPC1 disturbs metabolic homeostasis.

This article has an associated First Person interview with the first author of the paper.

## Abstract (precis)

**Summary:** TRPC1 modulates Ca^2+^ entry, which is essential in adipocyte differentiation and adiponectin secretion, through facilitating SNARE complex formation, thereby maintaining metabolic homeostasis.

## INTRODUCTION

Adipose tissue (AT) was initially considered an inert fat-storage depot until the discovery of linkages between obesity and inflammation (Rosen and Spiegelman, 2006). Currently, AT is considered a complex endocrine organ secreting over 600 bioactive factors (Lehr et al., 2012; MacDougald and Burant, 2007), termed adipokines. Adipokines influence diverse physiological processes by relaying information to other metabolically active organs such as muscle, liver, pancreas and brain, thereby modulating systemic metabolism (Ouchi et al., 2011). Importantly, increased adipose accumulation, as seen in obesity, correlates to dysregulation of adipokine secretion (Arita et al., 1999; Matsuzawa, 2010) and increases susceptibility to other diseases (Rasouli and Kern, 2008). Altering levels of the adipokines, mainly adiponectin (Fruebis et al., 2001; Okada-Iwabu et al., 2013) and leptin (Stern et al., 2016), has been shown to improve energy homeostasis and offset diet-induced obesity. Thus, adipokines have potential to be therapeutic targets for the treatment or reduction of obesity and other metabolic diseases; however, a full understanding of the mechanisms and intracellular mediators that modulate adipokine secretion within AT is first required.

Secreted, adiponectin occurs in three isoforms, a low or medium molecular weight trimer-dimer and an oligomeric complex of greater molecular weight, that modulate diverse physiological functions including thermogenesis (Hui et al., 2015) and sensitizing insulin signaling (Yamauchi et al., 2001). Regulated exocytosis in adipocytes mediates key functions, exemplified by insulin-stimulated secretion of peptides such as adiponectin, and recycling of intracellular membranes containing GLUT4 (also known as SLC2A4) glucose transporters to the cell surface (Bogan and Lodish, 1999). Release of adiponectin from adipocytes has been shown to be insulin- and Ca^2+^-dependent (Bogan and Lodish, 1999; Cong et al., 2007; Komai et al., 2014; Xie et al., 2008) and a high-Ca^2+^ diet has been shown to stimulate the expression of adipokines along with inhibition of pro-inflammatory factors (Sun and Zemel, 2007). However, the molecular mechanisms of these responses and their relationship with Ca^2+^ channels is not known. Adiponectin is initially synthesized as a pre-hormone, oligomerized into three isoforms and stored in vesicles until release is stimulated. Plasma membrane proteins SNAP-25 and syntaxin, termed t-SNAREs, and secretory vesicle-associated protein VAMP or v-SNARE, are part of the conserved protein complex involved in fusion of opposing membranes necessary for exocytosis. It has been demonstrated that Ca^2+^ is crucial to the final event needed for vesicle fusion and release of its content (Brose et al., 1992).

Several studies have shown that the anatomical distribution of adipose tissue dictates differing characteristics where subcutaneous adipocytes have distinct metabolic properties from visceral adipocytes (Baglioni et al., 2012). Proliferation of subcutaneous adipose tissue (Subc-AT) may be considered beneficial, in part, by increasing ‘healthy’ lipid storage capacity that produces fewer inflammatory cytokines. By contrast, visceral adipose tissue (VAT) is thought to be more inflammatory (Kadiri et al., 2017; O'Rourke et al., 2009) and leads to the development of obesity and related metabolic diseases (Foster et al., 2011; Seale et al., 2011). Although the debate about the metabolic function of Subc-AT and VAT is not yet settled (Fabbrini et al., 2010; Thörne et al., 2002), variances in distribution of lipids in Subc-AT and VAT could be critical in the development of metabolic diseases. One area to investigate regarding variances between Subc-AT and VAT tissues could be the process of differentiation of committed preadipocytes into mature adipocytes. Interestingly, it has been shown that differentiation is impaired in subcutaneous preadipocytes of obese individuals, and the ability of these cells to differentiate is negatively correlated to cell size and the individual's BMI (Isakson et al., 2009). Further, in an obese state, the preadipocyte to mature adipocyte ratio is reduced and the ability of Subc-AT to differentiate properly may be diminished, pushing the accumulation of fat to visceral depots (Tchoukalova et al., 2007). Similar results have shown an age-dependent decline in differentiation, and increases in adipose tissue accumulation in obesity is likely due to hypertrophy instead of hyperplasia (Kim et al., 2014). Although several nuclear receptors and transcription factors that regulate adipocyte differentiation and adipogenesis have been identified (Farmer, 2006), factors upstream to the activation of these transcription factors are still unknown. The activation of peroxisome proliferator-activated receptor γ (PPARγ) occurs in the intermediate or late stage of adipogenesis, and PPARγ activation has been determined to be a master regulator of adipogenesis (Haider et al., 2017). PPARγ has been linked to the transcription of genes expressed in mature adipocytes such as fatty acid binding protein (FABP4), required for transport of free fatty acids, and perilipin (PLIN1), which covers the surface of mature lipid droplets in adipocytes and regulates lipolysis. Calcium has been identified as being important for the transcriptional regulation of adipocyte differentiation; however, the molecular identity of the Са^2+^ channel has yet to be determined. Studies on preadipocytes show that elevating intracellular Ca^2+^ concentrations ([Ca^2+^]_i_) early in differentiation inhibits PPARγ induction and triglyceride accumulation (Neal and Clipstone, 2002).

Ca^2+^ is an important secondary messenger molecule involved in regulating numerous cell processes including cell proliferation and differentiation, gene transcription and exocytosis (Berridge et al., 2000), and is controlled by altering [Ca^2+^]_i_. Changes in [Ca^2+^]_i_ are the result of either the release of Ca^2+^ stored in the endoplasmic reticulum (ER) that initiates store-operated Ca^2+^entry (SOCE) (Parekh and Putney, 2005; Putney et al., 2017) or direct Ca^2+^ entry from the extracellular space mediated by various types of channels including voltage-gated Ca_V_ channels (Catterall, 2000) and channels belonging to the seven-member transient receptor potential canonical (TRPC) protein family (Birnbaumer et al., 1996). During SOCE activity, ER Ca^2+^ stores are depleted and the ER protein stromal interaction molecule 1 (STIM1) approaches the plasma membrane where it interacts with ORAI1 (Feske et al., 2006) and TRPC1 channels (Asanov et al., 2015; Liao et al., 2008), resulting in Ca^2+^ influx and increased [Ca^2+^]_i_. Microarray analysis has shown that both TRPC1 and TRPC5 expression are induced when adipocytes mature; however, the Ca^2+^ entry channel in subcutaneous and visceral adipocytes has yet to be physiologically characterized.

The aim of this study was to identify the endogenous Ca^2+^ entry channel in adipocytes and establish its physiological function in modulating adipocyte differentiation and adipokine secretion. We report for the first time the AT type-dependent differences in sensitivity to SOCE, differentiation, and lipid accumulation. We further report that the endogenous Ca^2+^ entry channel in adipocytes is dependent on TRPC1, and loss of TRPC1 function inhibits adipocyte differentiation. Furthermore, loss of TRPC1 inhibits vesicular fusion by SNARE proteins, and TRPC1^−/−^ mice exhibited increased adipose deposition along with having reduced serum adiponectin and leptin concentrations. Taken together, these results suggest that TRPC1 is essential for adipocyte differentiation and adipokine secretion, which regulate metabolic homeostasis to reduce obesity.

## RESULTS

### SOCE is essential for the differentiation of subcutaneous adipocytes

**Fig. 1.**
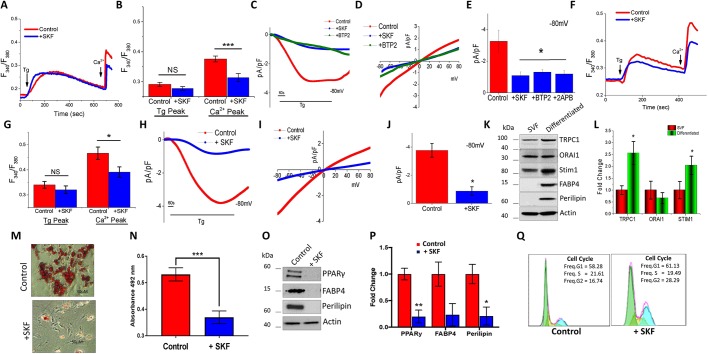
**SOCE is essential for the differentiation of subcutaneous adipocytes.** (A,B,F,G) Fura-2 fluorescence traces (F_340_/F_380_, 340 nm:380 nm fluorescence ratio) of transient increase in [Ca^2+^]_i_ after addition of 1 μM Tg and 1 mM Ca^2+^ to Subc-AT SVF (A) and differentiated Subc-AT (F) in control and cells pre-treated with 10 μM SKF for 15 min. Graphs quantify Tg-induced ER Ca^2+^ release and Ca^2+^ entry peaks for Subc-AT SVF cells (B) and differentiated Subc-AT (G). Each bar gives the mean±s.e.m. of 60–90 cells in three separate experiments. (C–E,H–J) Application of 1 μM Tg induced inward currents at −80 mV in control, SKF-, 2APB- or BTP-treated Subc-AT SVF cells (C) and differentiated Subc-AT (H). Respective I–V curves are shown for Subc-AT SVF (D) and differentiated Subc-AT (I). Quantification (*n*=7–9 recordings) of current intensity at −80 mV is shown for Subc-AT SVF (E) and differentiated Subc-AT (J). (K,L) Western blot (K) with quantification (L) of TRPC1, ORAI1, Stim1, FAPB4 and perilipin protein expression normalized to actin and presented as fold change relative to control untreated cells in Subc-AT SVF and differentiated Subc-AT (*n*=3). (M) Oil-Red-O staining (using 10× objective) of Subc-AT differentiated in the presence of SKF (10 µM) for 7 days. (N) Quantification of absorbance at 492 nm of stained lipid droplets using eluted Oil-Red-O stain (*n*=3). (O,P) Western blot (O) and protein level quantification (P, normalized to actin and presented as fold change relative to control untreated cells) of PPARγ, FAPB4 and perilipin protein expression in differentiated Subc-AT, in control cells and in the presence of SKF (*n*=3–4). (Q) Cell cycle analysis on control and SKF-treated Subc-AT cells (10 µM). Graphs are mean±s.e.m. *P<0.05, ***P<0.001 using one-way ANOVA.

To establish the molecular identity of the SOCE channel in adipose cells, we evaluated Ca^2+^ signaling in isolated stromal vascular fraction (SVF) of mouse AT. Addition of 1 µM thapsigargin (Tg), a sarcoplasmic/endoplasmic reticulum calcium ATPase (SERCA) pump blocker that causes loss of Ca^2+^ from the internal ER stores, resulted in a small increase in [Ca^2+^]_i_ levels (first peak) in Subc-AT SVF cells (Fig. 1A,B). In the presence of 1 mM external Ca^2+^, Subc-AT SVF cells showed a significant increase in [Ca^2+^]_i_ levels (second peak), indicating the presence of store-mediated Ca^2+^ entry (Fig. 1A,B). Importantly, Subc-AT SVF cells treated with 10 µM SKF96365 (SKF), a blocker of store-mediated Ca^2+^ influx channels, were observed to have a significant reduction in SOCE without any change in internal ER Ca^2+^ release (Fig. 1A,B). To establish the molecular identity of the Ca^2+^ influx channel in Subc-AT cells, electrophysiological recordings of membrane currents were performed. Addition of Tg induced an inward current, which was non-selective in nature and reversed between 0 and −5 mV (Fig. 1C,D). The current properties observed in Subc-AT SVF cells were similar to previous recordings observed with TRPC1 channels (Liu et al., 2004; Selvaraj et al., 2012; Shi et al., 2012; Yuan et al., 2007). Moreover, pretreatment with SKF, 2APB, a blocker of IP_3_ and TRP channels, or BTP2, a Ca^2+^ release-activated channel (CRAC) inhibitor, significantly inhibited the Tg-induced nonselective current (Fig. 1C–E), suggesting that in Subc-AT SVF cells, the SOCE is dependent on TRPC1 channels.

We next evaluated the physiological properties of the SOCE channels in differentiated adipocytes from Subc-AT. Subc-AT SVF cells were cultured and differentiated into mature adipocytes *ex vivo*. Similar to the observed response by Subc-AT SVF cells, differentiated subcutaneous adipocytes showed no change in the ER Ca^2+^ release (upon addition of 1 µM Tg) (Fig. 1F,G). Addition of 1 mM external Ca^2+^ also caused an increase in [Ca^2+^]_i_ levels, which was significantly higher than in non-differentiated cells (Fig. 1A,B), but was also reduced when cells were treated with SKF as compared to untreated differentiated control (Fig. 1F,G). Furthermore, a TRPC1-like current similar to that seen in undifferentiated cells was observed in differentiated Subc-AT adipocytes and the Tg-induced currents were again inhibited by SKF (Fig. 1H–J). Comparison of Subc-AT SVF to differentiated Subc-AT adipocytes indicated a ∼25% increase in amplitude of TRPC1-like currents in differentiated Subc-AT (Fig. 1E,J).

To identify the expression of calcium channels in modulating SOCE, protein expression of TRPC1, STIM1 and ORAI1 in both Subc-AT SVF and differentiated Subc-AT was analyzed. Consistent with other studies, expression of TRPC1 and STIM1 was increased upon differentiation (Graham et al., 2009; Sukumar et al., 2012); however, there was no change in ORAI1 expression (Fig. 1K,L). Known markers of adipocyte differentiation, FABP4 and perilipin, were used to confirm differentiation. Interestingly, altering [Ca^2+^]_i_ inhibits differentiation of adipocytes (Jensen et al., 2004), thus, we speculated whether blocking SOCE using SKF would have a similar effect. Subc-AT SVF adipocytes were differentiated in the presence of SKF (10 µM) for 7 days and lipid accumulation was detected by Oil-Red-O (Fig. 1M,N). Treatment with SKF significantly reduced the ability of Subc-AT SVF cells to accumulate intracellular lipids. In addition, SKF treatment reduced expression of differentiation markers FABP4, perilipin and PPARγ, further indicating the involvement of Ca^2+^ influx via SOCE channels in adipocyte differentiation (Fig. 1O,P). Interestingly, cell cycle was also affected and SKF treatment significantly increased the number of cells in G2-M phase (Fig. 1Q). Taken together, these results indicate that SOCE in Subc-AT is dependent on TRPC1 before and after differentiation and may have an important role in the mechanisms of differentiation and lipid accumulation.

### Differentiated visceral adipocytes exhibit increased in SOCE

**Fig. 2.**
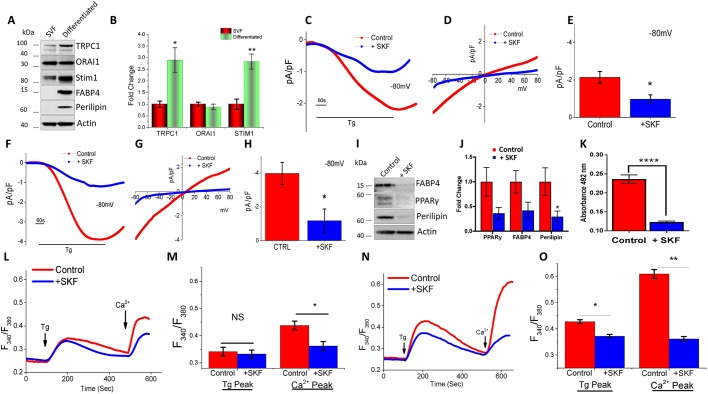
**Differentiated visceral adipocytes also exhibit increase in SOCE.** (A,B) Western blots (A) of TRPC1, ORAI1, Stim1, FAPB4 and perilipin, and actin protein expression, protein level quantification normalized to actin provided as bar graph (B), of VAT SVF and differentiated VAT (*n*=3). (C–H) Application of 1 μM Tg induced inward currents at −80 mV in control and SKF treated VAT-SVF cells (C) and differentiated VAT (F). Respective I–V curves are shown for VAT SVF (D) and differentiated VAT (G). Quantitation (*n*=6 recordings) of current intensity at −80 mV for VAT SVF (E) and differentiated VAT (H). (I,J) Western blot (I) and protein level quantification (J, normalized to actin and presented as fold change relative to control untreated cells) of PPARγ, FAPB4 and perilipin protein expression of differentiated VAT treated with SKF (*n*=3–4). (K) Quantification of eluted Oil-Red-O stain at 492 nm of differentiated VAT in the presence of SKF (10 µM) for 7 days (*n*=3). (L–O) Fura-2 fluorescence traces of transient increase in [Ca^2+^]_i_ after addition of 1 μM Tg and 1 mM Ca^2+^ to VAT SVF cells (L) and differentiated VAT (N) pretreated with 10 μM SKF for 15 min. Quantification of Tg and Ca^2+^ peaks for VAT SVF cells (M) and differentiated VAT (O). Each bar gives the mean±s.e.m. of 40–60 cells in three separate experiments. Graphs are mean±s.e.m. *P<0.05, **P<0.01, ****P<0.0001 using one-way ANOVA.

White AT has different properties based on its location. It is well known that VAT, when compared with Subc-AT, is functionally different in that VAT has higher inflammatory potential (Zhou et al., 2007) as it contains a greater number of inflammatory and immune cells. VAT has greater oxidative capacity and lipolysis potential than Subc-AT and less capacity to differentiate, resulting in a greater percentage of large adipocytes as compared to Subc-AT (Ibrahim, 2010). To date, it is unknown whether the store-mediated changes in [Ca^2+^]_i_ that lead to adipocyte differentiation are analogous between VAT and Subc-AT. Thus, we investigated SOCE from adipocytes derived from VAT depots. Consistent with Subc-AT protein expression, STIM1, ORAI1 and TRPC1 were expressed in both VAT SVF and differentiated VAT adipocytes, with increased expression of STIM1 and TRPC1 seen in differentiated VAT with no change in ORAI1 expression (Fig. 2A,B). Membrane current recordings of VAT SVF adipocytes induced upon the addition of 1 µM Tg showed an inward TRPC1-like current, and pretreatment with 10 µM SKF significantly inhibited the nonselective current (Fig. 2C–E). Importantly, after differentiation, a similar TRPC1-like current was observed in VAT cells (Fig. 2F–H) and a ∼50% increase in the current amplitude was observed upon differentiation (Fig. 2E,H). Again, Tg-induced currents were inhibited by the addition of SKF in differentiated VAT adipocytes; however, the residual current observed was similar to Ca^2+^ release-activated channel currents (*I*_CRAC_) (Fig. 2F–H). The effect of SKF on lipid accumulation was also evaluated in differentiating VAT SVF adipocytes with the addition of 10 µM SKF (Fig. 2K). Similar to Subc-AT, addition of SKF significantly reduced the ability of VAT to accumulate intracellular lipids as well as reduced protein expression of differentiation markers (Fig. 2I,J). Calcium imaging of VAT SVF adipocytes treated with SKF revealed no change in internal Ca^2+^ release, but a significant reduction in SOCE (Fig. 2L,M). However, differentiated VAT adipocytes treated with SKF showed a reduction in both internal Ca^2+^ ER store release upon Tg treatment, and SOCE upon addition of external Ca^2+^ (Fig. 2N,O). These results indicate that differentiated VAT adipocytes may be more sensitive to alterations in SOCE than VAT SVF adipocytes; however, the properties of the SOCE and role in differentiation and lipid accumulation are similar between Subc-AT and VAT.

### TRPC1^−/−^ mice have increased adiposity with age

**Fig. 3.**
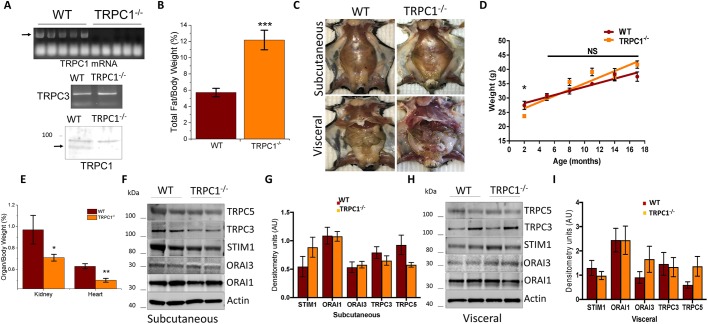
**TRPC1^−/−^ mice have increased adiposity with age.** (A) mRNA expression of TRPC1 and TRPC3 from Subc-AT of WT and TRPC1^−/−^ mice, and western blot of TRPC1 protein expression. (B) Body fat percentage calculated by dividing the fat weight by total body weight of mice aged 13–15 months (*n*=9–11). (C) Exposed Subc-AT and Visc AT of WT and TRPC1^−/−^ male mice aged 9 months. (D) Total body weight of WT and TRPC1^−/−^ mice by 3-month age groups (*n*=8–15 for each age). (E) Ratio of organ to total body weight of WT and TRPC1^−/−^ mice (*n*=6–10). (F–I) Western blot and protein level quantification (normalized to actin) of TRPC5, TRPC3, STIM1, ORAI3 and ORAI1 protein expression in Subc-AT (F,G) and VAT (H,I) tissue lysates (*n*=6–8). Graphs are mean± s.e.m. *P<0.05, **P<0.01, ***P<0.001 either using Student's *t*-test or one-way ANOVA.

Both the increase in TRPC1 expression upon adipocyte differentiation and the reduction in SOCE observed when TRPC1 is blocked led us to investigate whether dysfunctional TRPC1 channels could alter overall body composition. Utilizing homozygous TRPC1 knockout mice (TRPC1^−/−^), we confirmed the absence of TRPC1 in Subc-AT by assessing TRPC1 mRNA and protein expression, but did not observe any change in TRPC3 mRNA (Fig. 3A). Body mass composition analysis was performed on TRPC1^−/−^ and wild-type (WT) mice aged 13–15 months, which resulted in a more than twofold increase in the ratio of total fat to body weight in TRPC1^−/−^ mice as compared to WT mice (Fig. 3B). Visually, this difference in body composition can be seen in Fig. 3C where TRPC1^−/−^ mice have increased Subc-AT and VAT volume as compared to WT counterparts. Interestingly, the increase in total fat observed in TRPC1^−/−^ mice did not translate to an increase in their overall body weight. Within a majority of age groups, no significant difference in the overall body weight was observed in TRPC1^−/−^ when compared with WT mice; however, in TRPC1^−/−^ mice there seems to be a greater trend of age-related increased body mass (Fig. 3D). Further, no difference in food consumption nor lean body mass or skeletal muscle mass was observed (data not shown). TRPC1^−/−^ mice did, however, have lower organ weights (such as hearts and kidneys), which were reduced in size regardless of age and may contribute to the increase in adiposity without an increase in overall body weight (Fig. 3E). Protein expression analysis of numerous Ca^2+^-regulating proteins in Subc-AT and VAT was also performed to rule out compensation for the lack of TRPC1. As shown in Fig. 3F–I, no change in channel protein expression was observed between WT and TRPC1^−/−^ AT, indicating that the lack of TRPC1 function in AT most likely does not change protein expression of other Ca^2+^ channels. Taken together, these data suggest that the loss of TRPC1 function contributes to an increase in the accumulation of adipocytes, but not overall weight. Furthermore, the loss of TRPC1 function is not compensated by other TRPCs and ORAIs in AT.

### Loss of TRPC1 in adipocytes reduces SOCE and ability to differentiate

**Fig. 4.**
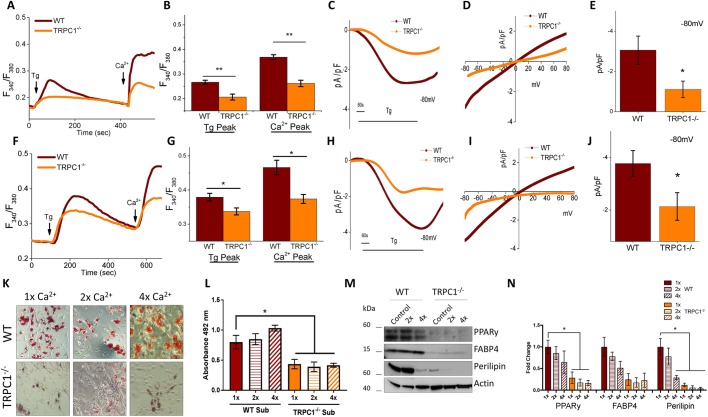
**Loss of TRPC1 in subcutaneous adipocytes reduces SOCE and adipogenesis.** (A,B,F,G) Fura-2 fluorescence traces of transient increase in [Ca^2+^]_i_ after addition of 1 μM Tg and 1 mM Ca^2+^ to WT and TRPC1^−/−^ Subc-AT SVF (A) and differentiated Subc-AT (F). Bar graph quantifies Tg and Ca^2+^ peaks for Subc-AT SVF cells (B) and differentiated Subc-AT (G). Each bar gives the mean±s.e.m. of 40–60 cells in three separate experiments. (C–E,H–J) Application of 1 μM Tg induced inward currents at −80 mV in WT and TRPC1^−/−^ Subc-AT SVF (C) and differentiated Subc-AT (H). Respective I–V curves of Subc-AT SVF (D) and differentiated Subc-AT (I). Quantification (*n*=5 recordings) of current intensity at −80 mV is shown for Subc-AT SVF (E) and differentiated Subc-AT (J). (K) Oil-Red-O staining (using 10× objective) of WT and TRPC1^−/−^ Subc-AT differentiated in basal (1×), twofold (2×) or fourfold (4×) levels of extracellular Ca^2+^ for 7 days. (L) Quantification of eluted Oil-Red-O stain at 492 nm (*n*=7). (M,N) Western blot (M) and protein level quantification (N, normalized to actin and presented as fold change relative to control untreated cells) of PPARγ, FAPB4 and perilipin protein expression in WT and TRPC1^−/−^ Subc-AT differentiated in basal, twofold or fourfold levels of extracellular Ca^2+^ (*n*=3). Graphs are mean±s.e.m. *P<0.05, **P<0.01 using one-way ANOVA.

To confirm that the loss of TRPC1 negatively effects SOCE fluctuations within Subc-AT and VAT, SVF cells from TRPC1^−/−^ and WT mice were isolated and differentiated into adipocytes. Analysis by Ca^2+^ imaging of both Subc-AT cell types (SVF and differentiated adipocytes) showed that TRPC1^−/−^ cells undergo reduction in internal ER Ca^2+^ release (first peak) with the addition of Tg as compared to WT (Fig. 4A,B,F,G). When external Ca^2+^ was added, [Ca^2+^]_i_ in both SVF and differentiated TRPC1^−/−^ cells was significantly reduced, indicating diminished SOCE in TRPC1^−/−^ cells. Consistent with these results, TRPC1 currents were significantly reduced in Subc-AT SVF in TRPC1^−/−^ mice (Fig. 4C–E). Similar results were observed in differentiated Subc-AT, where loss of TRPC1 significantly decreased Tg-mediated Ca^2+^ currents (Fig. 4H–J). As shown in Figs 1 and 2, modifying [Ca^2+^]_i_ affects adipocyte differentiation. Thus, we investigated whether a lack of TRPC1 would alter the ability of TRPC1^−/−^ Subc-AT SVF cells to differentiate. Analysis of lipid accumulation in WT and TRPC1^−/−^ Subc-AT after differentiation revealed a significant reduction in total lipid when TRPC1 is knocked down (Fig. 4K,L). Protein expression levels of perilipin, FABP4 and PPARγ were also reduced in TRPC1^−/−^ Subc-AT as compared to WT, with PPARγ and perilipin reduced to a significant degree (Fig. 4M,N). To offset the reduced [Ca^2+^]_i_ of TRPC1^−/−^ cells, Subc-AT SVF cells from WT and TRPC1^−/−^ mice were differentiated with a two- and fourfold greater extracellular Ca^2+^ concentration. In WT and TRPC1^−/−^ Subc-AT cells, increasing extracellular Ca^2+^ by both two- and fourfold produced no change in lipid accumulation; however, TRPC1^−/−^ adipocytes had significantly less lipid than WT. The increase in extracellular Ca^2+^ concentration by two- and fourfold resulted in a decrease in expression of perilipin, FABP4 and PPARγ in both WT and TRPC1^−/−^ subcutaneous adipocytes. Taken together, this data indicates that TRPC1 is an important SOCE channel in Subc-AT and loss of TRPC1 results in reduced Ca^2+^ influx and diminished differentiation. Furthermore, extracellular Ca^2+^ concentrations are important in adipogenic protein expression.

**Fig. 5.**
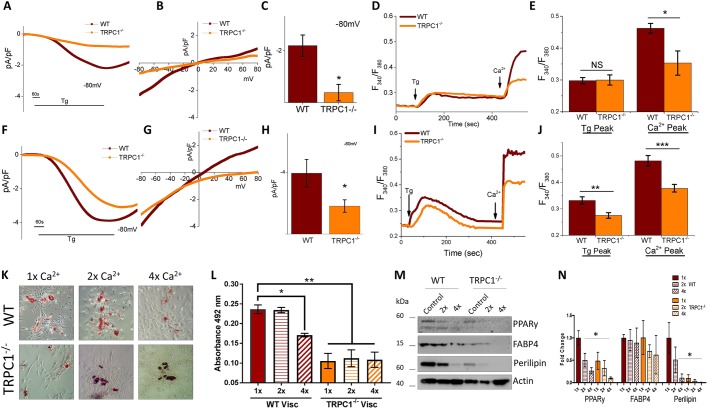
**Visceral adipocytes from TRPC1^−/−^ mice have reduced SOCE and ability to differentiate.** (A–C,F–H) Application of 1 μM Tg induced inward currents at −80 mV in WT and TRPC1^−/−^ VAT SVF (A) and differentiated Subc-AT (F). Respective I–V curves shown for VAT SVF (B) and differentiated VAT (G). Quantitation (*n*=8 recordings) of current intensity at −80 mV for VAT SVF (C) and differentiated VAT (H). (D,E,I,J) Fura-2 fluorescence traces of transient increase in [Ca^2+^]_i_ after addition of 1 μM Tg and 1 mM Ca^2+^ to WT and TRPC1^−/−^ VAT SVF (D) and differentiated VAT (I). Bar graph quantifies Tg and Ca^2+^ peaks for VAT SVF (E) and differentiated VAT (J). Each bar gives the mean±s.e.m. of 40–60 cells in three separate experiments. (K) Oil-Red-O staining (using 10× objective) of WT and TRPC1^−/−^ VAT differentiated in basal, twofold or fourfold levels of extracellular Ca^2+^ for 7 days. (L) Quantification of eluted Oil-Red-O stain at 492 nm (*n*=4). (M,N) Western blot (M) and protein level quantification (N, normalized to actin and presented as a fold change relative to control untreated cells) of PPARγ, FAPB4 and perilipin protein expression of WT and TRPC1^−/−^ VAT differentiated in basal, twofold or fourfold levels of extracellular Ca^2+^ (*n*=3). Graphs are mean±s.e.m. *P<0.05, **P<0.01, ***P<0.001 using two-way ANOVA.

Next, VAT from WT and TRPC1^−/−^ mice was evaluated in the same manner as for Subc-AT experiments. Electrophysiological recordings of membrane currents indicate that TRPC1 currents were significantly reduced in both VAT SVF TRPC1^−/−^ cells (Fig. 5A–C) and differentiated TRPC1^−/−^ VAT (Fig. 5F–H). Interestingly, after differentiation, the Ca^2+^ current in TRPC1^−/−^ VAT was more inwardly rectifying and showed properties similar to ORAI1 currents. Analysis of Ca^2+^ influx upon treatment with Tg showed a decrease in internal ER Ca^2+^ release in TRPC1^−/−^ differentiated VAT adipocytes but not in undifferentiated VAT SVF (Fig. 5D,E,I,J). Similar to Subc-AT, addition of external Ca^2+^ resulted in a reduction in Ca^2+^ influx in both SVF and differentiated TRPC1^−/−^ VAT as compared to WT control. Analysis of lipid accumulation in WT and TRPC1^−/−^ VAT after differentiation revealed a significant reduction in total lipid when TRPC1 is knocked down (Fig. 5K,L). This lack of differentiation in TRPC1^−/−^ VAT adipocytes was also evident in the lack of expression of perilipin, FABP4 and PPARγ (Fig. 5M,N). Notably, increasing extracellular Ca^2+^ concentration during differentiation decreased lipid accumulation when increased by fourfold in WT VAT; however, no change was observed in TRPC1^−/−^ VAT (Fig. 5K,L). When challenged with increased extracellular Ca^2+^ concentrations, expression of perilipin, FABP4 and PPARγ progressively decreased in both WT and TRPC1^−/−^ VAT (Fig. 5M,N). Taken together, these results reveal TRPC1 to be a functioning partner in SOCE in not only Subc-AT but also VAT. Differentiation in both Subc-AT and VAT is inhibited by increased extracellular Ca^2+^ concentrations; however, Subc-AT may be less sensitive as shown by total lipid accumulation. Further, differentiation in both tissue types, Subc-AT and VAT, is impaired when TRPC1 is knocked down.

### Serum adipokine levels are reduced in TRPC1^−/−^ mice

**Fig. 6.**
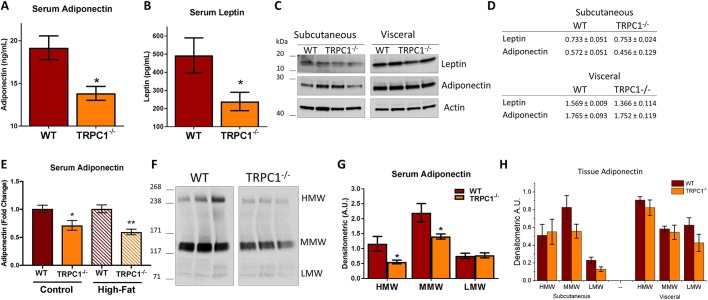
**Serum adipokines and isoforms are altered in TRPC1^−/−^ mice.** (A,B) ELISA-assessed total serum adiponectin (A, *n*=6) and leptin (B, *n*=8) of WT and TRPC1^−/−^ mice aged 3–9 months. (C,D) Western blot (C) and protein level quantification (D) of leptin and adiponectin protein expression normalized to actin of WT and TRPC1^−/−^ tissue lysates of Subc-AT and VAT (*n*=3). (E) ELISA-assessed total serum adiponectin from WT and TRPC1^−/−^ mice fasted overnight after 12 weeks on normal chow (16% fat) or high fat (45% fat) (*n*=8) and presented as a fold change relative to control untreated cells. (F,G) Serum adiponectin isoforms in WT and TRPC1^−/−^ mice assessed by non-denaturing PAGE (F). Optical density of each isoform (HMW, high molecular weight; MMW, medium molecular weight; LMW, low molecular weight) normalized to total protein from respective Ponceau S stains and quantified (G) (*n*=4). (H) Tissue adiponectin isoforms in WT and TRPC1^−/−^ Subc-AT and VAT protein tissue lysates assessed by non-denaturing PAGE (not shown) and quantified (*n*=4). Graphs are mean±s.e.m. *P<0.05, **P<0.01 either using Student's *t*-test or two-way ANOVA. A.U., arbitrary units.

Ca^2+^ has been shown in numerous studies to be involved in the secretion of adipokines from AT (Komai et al., 2014; Sukumar et al., 2012; Ye et al., 2010); however, the Ca^2+^ channel(s) involved have not been identified. To determine whether the reduction in Ca^2+^ influx in TRPC1^−/−^ AT alters adipokine levels, we assessed serum adiponectin and leptin concentrations in WT and TRPC1^−/−^ mice. Serum concentrations of both adiponectin and leptin were significantly reduced in TRPC1^−/−^ mice aged 3–9 months as compared to their WT age-matched counterpart, with no difference seen at any age group (i.e. the age of the mouse made no difference to adiponectin levels). (Fig. 6A,B). There are conflicting views on the effect of a high-fat diet on serum adiponectin concentrations (Bullen et al., 2007), which led us next to investigate whether a high-fat diet would alter serum adipokine concentrations in TRPC1^−/−^ mice. WT and TRPC1^−/−^ mice were placed on a diet of either normal chow (16% fat) or high fat (45% fat) for 12 weeks and serum adiponectin concentrations were assessed at the end of 12 weeks. Results show a reduction in serum adiponectin concentrations in TRPC1^−/−^ mice fed a normal chow diet when compared to WT; however, the high-fat diet failed to induce a change in serum adiponectin concentrations in the TRPC1^−/−^ mice (Fig. 6E).

Adiponectin and leptin are almost entirely produced by adipocytes, which led us to question whether the reduction in serum concentrations seen in TRPC1^−/−^ mice was due to a decrease in overall production. To assess this, Subc-AT and VAT lysates from WT and TRPC1^−/−^ mice were evaluated for adiponectin and leptin protein expression. No difference was observed in either adiponectin or leptin expression between AT obtained from TRPC1^−/−^ or their WT counterparts (Fig. 6C,D). Interestingly, both WT and TRPC1^−/−^ VAT samples contained higher levels of both adiponectin and leptin than Subc-AT samples, possibly indicating that VAT may play a larger role as a distributor of adipokines. Overall, these results suggest that reduced serum adiponectin and leptin concentrations in TRPC1^−/−^ mice are probably not a consequence of lower AT production, and that TRPC1^−/−^ mouse serum adiponectin concentrations are not affected by a high-fat diet.

### Adiponectin isoforms are altered in TRPC1^−/−^ mice

Adiponectin is first formed as a 30 kDa monomer that can be assembled within the ER into complex isoforms such as high (HMW), medium (MMW), and low (LMW) molecular weights. The HMW isoform is thought to be the most biologically active and Ca^2+^ is important in its formation (Banga et al., 2008; Rosen and Spiegelman, 2014). Though Ca^2+^ dependency has been shown, the specific Ca^2+^ channel involved has yet to be determined. Evaluation of serum adiponectin from WT and TRPC1^−/−^ mice by non-denaturing gel electrophoresis indicated a significant reduction in both HMW and MMW isoforms with no difference in LMW (Fig. 6F,G). To understand whether the reduction in HMW and MMW isoforms in TRPC1^−/−^ mouse serum is due to an issue in adiponectin production and folding within AT, Subc-AT and VAT adiponectin isoform composition was assessed. In all three isoforms, there was no difference observed between WT and TRPC1^−/−^ in either Subc-AT or VAT, indicating that isoform assembly is not a likely candidate for differences in serum levels (Fig. 6H). Within these studies, we observed that TRPC1^−/−^ mice have less biologically active adiponectin (HMW) in serum than WT mice (Fig. 6F,G), which does not correspond to intracellular tissue quantities (Fig. 6H).

### Reduced SNARE protein interactions in TRPC1^−/−^ adipose tissue

**Fig. 7.**
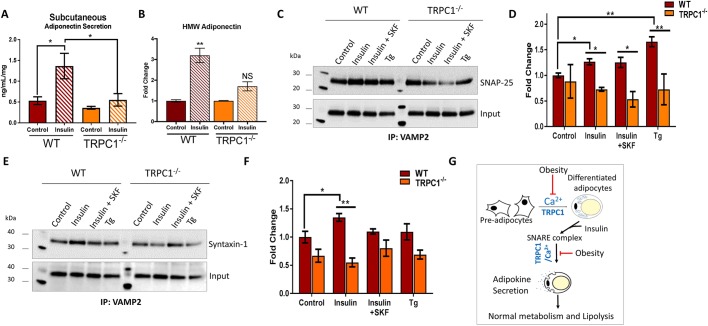
**Reduced SNARE protein interactions in TRPC1^−/−^ adipose tissue.** (A) ELISA-assessed total adiponectin secreted diponectin secreted from WT and TRPC1^−/−^ Subc-AT stimulated with 100 nM insulin for 6 h (*n*=8). (B) Quantification of HMW adiponectin isoform secreted in A assessed by non-denaturing PAGE (not shown), normalized to total protein from respective Ponceau S stains, quantified (*n*=4) and presented as a fold change relative to control untreated cells. (C–F) Co-immunoprecipitation assay of SNARE complexes in Subc-AT and VAT lysates from WT and TRPC1^−/−^ mice stimulated with 100 nM insulin and/or 10 μM SKF, or 1 μM Tg. Western blot analysis was performed using anti-VAMP2 antibody after immunoprecipitating with anti-SNAP25 (C, quantification in D, normalized to input and presented as a fold change relative control untreated cells) and anti-syntaxin-1 (E, quantification in F, normalized to input and presented as a fold change relative to control untreated cells) antibodies (*n*=3). Graphs are mean±s.e.m. *P<0.05, **P<0.01 using two-way ANOVA. (G) Proposed model where TRPC1 regulates obesity by initiating adipose differentiation and adipokine secretion required for normal metabolism.

Secretion of adiponectin from AT can be triggered through treatment with insulin. To mimic this process *ex vivo*, fresh Subc-AT from WT and TRPC1^−/−^ mice was cut into 8–15 mg pieces and stimulated with 100 nM insulin for 6 h. Upon insulin stimulation, secreted adiponectin concentrations were significantly increased in WT Subc-AT compared to non-treated control (Fig. 7A). Importantly, treatment of TRPC1^−/−^ Subc-AT with insulin did not result in a significant increase in adiponectin secretion as seen in the WT Subc-AT (Fig. 7A,B). Analysis of adiponectin isoforms upon insulin stimulation showed a reduction in HMW secretion (Fig. 7B) with a converse increase in LMW in all tissue types and animals (data not shown). These data indicate that adipocytes with TRPC1 deficiency has a reduced response to insulin-stimulated secretion of adiponectin.

Our lab has previously shown that a functional interaction between SNARE family proteins is required for intracellular vesicle fusion and Ca^2+^ influx (Singh et al., 2004). Vesicle-associated membrane protein 2 (VAMP2) is found within the plasma membrane and intracellular vesicles and is involved in the docking of vesicles to the plasma membrane by interacting with plasma membrane SNARE proteins, such as syntaxin and SNAP. To assess whether TRPC1 may be involved in SNARE complex formation needed to exocytose adipokine vesicles from AT, co-immunoprecipitation was performed on fresh AT samples. Subc-AT and VAT were isolated from WT and TRPC1^−/−^ mice and homogenized, quantified and incubated with insulin (100 nM), SKF (10 µM) or Tg (1 µM) for 30 min. Fig. 7C,E show that plasma membrane SNARE components SNAP25 and syntaxin 1 co-immunoprecipitated with VAMP2 in both WT and TRPC1^−/−^ control samples. Upon treatment with insulin, the interaction of VAMP2 with syntaxin 1 and SNAP25 increased in WT samples; however, no change was observed in TRPC1^−/−^. Pretreatment with SKF prior to insulin treatment did not change the effect of insulin on interactions of SNAP25 with VAMP2 in WT AT; however, it did reduce interactions of VAMP2 with syntaxin 1. Treatment of TRPC1^−/−^ AT cells with SKF had no effect on interactions of either syntaxin 1 or SNAP25 with VAMP2 (Fig. 7C–F). Raising [Ca^2+^]_i_ through treatment with Tg increased SNAP25 and VAMP2 interactions in WT AT; however, it did not result in a change in WT VAMP2 and syntaxin 1 interactions or any interactions within TRPC1^−/−^ AT. Taken together, these results indicate that insulin-stimulated SNARE complex formation in AT involves TRPC1-mediated Ca^2+^ entry, which is needed for exocytosis of adiponectin.

## DISCUSSION

The incidence of obesity-associated diseases such as type 2 diabetes, hypertension, cardiovascular risk and cancer has increased drastically worldwide. Consequently, scientific inquiry into the mechanisms underlying AT development and the pathology of obesity has gained interest. Though an array of metabolic disorders are linked to alterations in Ca^2+^ homeostasis (Arruda and Hotamisligil, 2015), the study of Ca^2+^ within AT has been underrepresented. Evidence suggests AT [Ca^2+^]_i_ is partly mediated by SOCE, thus, we focused on the identification of the endogenous Ca^2+^ entry channel and sought to establish its role in adipocyte function. In our study, we identify TRPC1 as a major regulator of adipocyte energy metabolism through mediation of adipocyte differentiation and SNARE complex formation needed for adipokine secretion.

SOCE was first identified as a major component of non-excitable cells, but further research has identified SOCE within a multitude of tissue types (Parekh and Putney, 2005). This knowledge has revealed it to be a ubiquitous Ca^2+^ signaling pathway that regulates numerous cellular functions including those connected with diabetic complications (Chaudhari and Ma, 2016). Identification of SOCE mechanisms within adipocytes may lead to a better understanding of the onset of obesity and metabolic disorders and reveal therapeutic possibilities. Within our study, Ca^2+^ entry was demonstrated to have SOCE properties that could effectively be reduced by the addition of non-specific Ca^2+^ entry channel blocker SKF in both Subc-AT and VAT, indicating a similar mechanism for Ca^2+^ entry influx between the tissue types. Furthermore, the current properties observed and blocked by SKF, 2APB and BTP2 were similar to those of the TRPC1 channel (Liu et al., 2003; Selvaraj et al., 2012). Upon differentiation, a higher amplitude of TRPC1-like current in both Subc-AT and VAT was observed, which is most likely attributable to the more than twofold upregulation of TRPC1 expression. The role of SOCE and the specific Ca^2+^ channel involved within Subc-AT and VAT was further confirmed using adipose tissue with a genetic ablation of TRPC1. Loss of TRPC1 was shown to cause a significant decrease in Ca^2+^ entry upon treatment with Tg in SVF and differentiated adipocytes of both AT types. In all TRPC1^−/−^ AT types except differentiated VAT, no change was observed in ER Ca^2+^ release in SKF-treated cells with the addition of Tg. The reduction of ER Ca^2+^ release in SKF-treated differentiated VAT is most likely attributed to a smaller ER Ca^2+^ pool and possibly a smaller ER. Although more research is needed to confirm, a smaller ER in TRPC1^−/−^ differentiated VAT could impair the ability of the ER to synthesize proteins and lipids properly. Importantly, although ORAI1 was present, it was not able to compensate for the loss of TRPC1, suggesting that the major Ca^2+^ entry channel in adipocytes is mediated via TRPC1. TRPC5 expression is increased upon adipocyte differentiation; however, as TRPC1 forms TRPC1–TRPC5 heterotetramers (Shi et al., 2012), loss of TRPC1 would inactivate TRPC5 and thus, TRPC5 could not compensate in TRPC1^−/−^ mice.

Adipocyte differentiation is a complex process and dysregulation can lead to lipid accumulation and altered energy and metabolic regulation. Although the process is not fully understood, several studies have identified a Ca^2+^ dependency within adipocyte differentiation (Jensen et al., 2004; Neal and Clipstone, 2002; Pramme-Steinwachs et al., 2017; Shi et al., 2000). Our studies are the first to use both genetic and pharmacological inhibitors to show that Ca^2+^ entry via a TRPC1-mediated channel is essential for adipocyte differentiation. Use of SKF to block store-mediated Ca^2+^ entry during differentiation not only impaired lipid droplet formation in WT Subc-AT and VAT SVF, but also inhibited the expression of lipid mobilization proteins FABP4 and perilipin. The transcription factor PPARγ is essentially expressed during early differentiation stages and regulates a number of adipocyte marker genes, including those encoding FABP4 and perilipin (Farmer, 2006; Wu et al., 1999). Here, we show that blockage of Ca^2+^ influx by SKF suppresses PPARγ expression, indicating that Subc-AT and VAT SVF may be unable to induce the initial processes of differentiation when SOCE is inhibited. Suppression of SOCE by TRPC1 knockdown, as seen in TRPC1^−/−^ Subc-AT and VAT cells, resulted in a reduction but not a complete impairment of lipid accumulation in both tissue types. Correspondingly, TRPC1^−/−^ Subc-AT and VAT differentiated adipocyte expression of FABP4, perilipin and PPARγ was reduced when compared to WT, indicating that TRPC1 may be involved in the initial stages of PPARγ-mediated differentiation. TRPC1 channels are also activated by G-protein-coupled receptors (GPCRs) that induce the production of second messenger inositol 1,4,5-trisphosphate (IP_3_), which bonds to IP_3_ receptors in the ER, thereby leading to store depletion. Recently, several GPCR genes have been identified in adipocyte cells (Klepac et al., 2016). For example endothelin receptors type A and B (*Ednra* and *Ednrb*), adrenoceptor alpha 1A (*Adra1a*), type-1A and type-1B angiotensin II receptor (*Agtr1a* and *Agtr1b*) and muscarinic acetylcholine receptor M3 (*Chrm3*). Importantly, an increase in the expression of GPCR genes has also been observed during differentiation, which further supports our data, and an increase in Ca^2+^ entry was observed in differentiated cells. In addition, insulin receptor signaling could also be involved. Binding of insulin to its receptor has been shown to inhibit SERCA, thus increasing cytosolic Ca^2+^ levels (Borge et al., 2002) and thus could also play a role in the activation of TRPC1 channels.

In contrast to Ca^2+^ blockage, continuous exposure to high external Ca^2+^ has been shown to inhibit preadipocyte differentiation (Jensen et al., 2004) and block adipocyte lipid accumulation and expression of adipogenic transcription factors (Pramme-Steinwachs et al., 2017; Shi et al., 2000). Within our study, increasing extracellular Ca^2+^ concentrations by fourfold decreased differentiation in WT VAT but had no effect on Subc-AT. Interestingly, a slight difference in lipid accumulation was observed between WT Subc-AT and WT VAT at all Ca^2+^ concentrations. Subc-AT is known to have greater differentiation potential and proliferative ability when compared to VAT (Baglioni et al., 2012), which most likely explains the differences observed in WT Subc-AT and VAT lipid accumulations. Subc-AT adipocytes were able to overcome the inhibitory effect of the increased extracellular Ca^2+^ to sustain lipid accumulation whereas VAT adipocytes were not. Although increasing extracellular Ca^2+^ concentrations had no effect on lipid accumulation in Subc-AT, protein expression of key differentiation factors reduced when Ca^2+^ increased, similar to the response observed in VAT. This may confirm what other studies have shown; that extended exposure to high Ca^2+^ concentrations inhibits differentiation of adipocytes, particularly in VAT. In addition, current properties in differentiated VAT cells, especially in TRPC1^−/−^ cells, were similar to the current properties of ORAI1. This suggests that ORAI1 could play a role in VAT tissues; however, further research is needed to fully establish the role of ORAI1 in these cells. Nonetheless, ORAI1 was not able to compensate for the loss of TRPC1.

Impaired adipogenesis can lead to a multitude of different outcomes, with the most common being an unhealthy expansion of adipose tissue. This phenomenon is generally seen in adult obesity, which is hypertrophic in nature and the reason why PPARγ agonists such as thiazolidinediones (TZDs) are useful in treating obesity (Choe et al., 2016). Within our study, TRPC1^−/−^ mice exhibited increased adipose tissue accumulation as they aged; however, overall body weight did not significantly increase relative to WT mice. This increase in adipose accumulation could be due to dysfunctional adipose differentiation and/or induction of lipogenesis, which in time results in a hypertrophic expansion. Another important finding was that organ weights were reduced, which could result from the critical role that TRPC1 has been shown to play in cell proliferation (Alonso-Carbajo et al., 2017) and be a reason for the observed increase in adipose accumulation without an associated increase in body weight. TRPC1^−/−^ mice could also have reduced lipolysis. If this is the case, fatty acids are not mobilized from triacylglycerol stores properly, which in turn would expand adipose tissue volume over time. Furthermore, deletion of FABP4 has been shown to reduce lipolysis and FABP4 knockout mice have increased white AT mass on a high-fat diet (Hotamisligil et al., 1996). In our model, lack of TRPC1 may inhibit the expression FABP4, thus reducing lipolysis. Another possibility is that TRPC1 could be involved in the store-operated Ca^2+^ entry necessary to induce lipolysis. Lack of store-operated Ca^2+^ entry proteins has been shown to disrupt regulators of lipid metabolism and result in increased organ lipid droplet formation (Maus et al., 2017). Taken together, these results indicate that SOCE via TRPC1 may be involved in the initial stages of adipocyte differentiation of both Subc-AT and VAT and result in increased adipose tissue volume over time; however, more research needs to be done to determine the exact mechanism.

Reduced secretion of adiponectin, resulting in low serum concentrations, is a common feature of obesity (Kovacova et al., 2012), whereas low serum leptin is used as an indicator of malnutrition (Amirkalali et al., 2010). TRPC1^−/−^ mice present both reduced serum adiponectin and leptin concentrations; however, TRPC1^−/−^ mice do not display either of these phenotypes. Food consumption and weight measurements of TRPC1^−/−^ mice were similar to WT throughout their lifetime; however, as they age their volume of adipose deposits increases. This increase in adiposity later in life is most likely due to suppressed adiponectin secretion, which leads to lower overall body metabolism and possible lipolysis dysfunction (Fig. 7G). Importantly, equal concentrations of adiponectin and leptin were observed within the Subc-AT and VAT samples of WT and TRPC1^−/−^ mice, which is contrary to the reduced serum concentrations in TRPC1^−/−^ mice. This indicates that there is no reduction in adipokine production, but rather the release of adipokines is affected in TRPC1^−/−^ mice. Interestingly, the greatest reduction in serum adiponectin in obese subjects is from Subc-AT and the metabolically active isoform HMW (Kovacova et al., 2012). Analysis of serum adiponectin isoforms in our study indicates a similar reduction in HMW secretion; however, internal AT concentrations of adiponectin were similar between WT and TRPC1^−/−^ mice. This reduction in TRPC1^−/−^ serum adiponectin HMW isoform most likely results from increased utilization by downstream targets, such as muscle and liver, since overall abundance is diminished. However, another caveat is that these are global TRPC1 knockouts that might affect additional systems, and caution should be used in the interpretation of the data. To understand the mechanisms of how TRPC1 regulates adipokine secretion, we provide evidence that insulin-stimulated TRPC1-mediated Ca^2+^ influx is necessary for fusion of VAMP2 with SNAP25 and syntaxin 1, and is thus necessary for the secretion of adipokines. Overall, our results provide evidence that TRPC1 not only plays a key role in adipocyte differentiation, but is essential for adipokine secretion, and dysfunction in these vital processes leads to obesity and metabolic syndrome.

## MATERIALS AND METHODS

### Animals

Male B6129SF2/J (WT) or TRPC1 knockout in the same background (TRPC1^−/−^) mice (Jackson Laboratories) were used for these experiments. All animals were housed in a temperature-controlled room under a 12 h:12 h light:dark cycle with *ad libitum* access to food and water. All animal experiments were carried out as per the institutional guidelines for the use and care of animals. For high-fat diet experimentation, 4-month-old males were fed diets containing either 16% (normal fat, NF) or 45% fat (high fat, HF) for 12 weeks. All animal protocols were approved by the institutional IACUC committee.

### Cell culture and cell cycle analysis

Subcutaneous and visceral adipose tissue was digested and SVF fraction was isolated for culture and differentiation as previously described (Claycombe et al., 2016; Harkins et al., 2004). Cell-cycle analysis was performed by staining the DNA with fluorescent dye (50 µg/ml propidium iodide) overnight, and data was analyzed using a flow cytometer.

### Calcium imaging

Cells were incubated with 2 µM Fura-2 (Molecular Probes) and the fluorescence intensity was monitored with a CCD camera-based imaging system (Compix) mounted on an Olympus XL70 inverted microscope equipped with an Olympus 40× (1.3 NA) objective. A dual wavelength monochromator enabled alternative excitation at 340 and 380 nm, whereas the emission fluorescence was monitored at 510 nm with an Orca imaging camera (Hamamatsu). Fluorescence traces shown represent [Ca^2^+]_i_ values in a 340 nm:380 nm ratio that are a representative of results obtained in at least three to four individual experiments using 40–70 cells in each experiment.

### Immunoblotting and co-immunoprecipiation

Crude lysates were prepared from Subc-AT and VAT, and SVF and differentiated adipocyte cultures. Protein was resolved on NuPAGE Novex 4–12% Bis-Tris gels, transferred to nitrocellulose membranes and probed with respective antibodies (see Table S1). Densitometric analysis was performed using ImageJ analysis and results were corrected for protein loading by normalization to β-actin levels. Non-denaturing PAGE was performed by resolving protein in Novex Tris-glycine native sample buffer on NuPAGE 3–8% Tris-acetate protein gels. For the co-immunoprecipiation assays, Subc-AT and VAT was manually homogenized in the presence of HBSS containing Ca^2+^ and Mg^2+^ and then treated as described in the paper, with 100 nM insulin and/or 10 μM SKF, or 1 μM Tg for 30 min at 37°C. Samples were incubated overnight with anti-VAMP2 antibody after which Pierce Protein A-Agarose IgG beads were added. Isolated beads were then washed, boiled and proteins separated by SDS-PAGE. SNARE proteins were detected using the indicated antibodies.

### EchoMRI measurements of body composition

Whole body composition, including fat mass and lean mass, was determined using nuclear magnetic resonance technology with an EchoMRI700 instrument (Echo Medical Systems).

### Oil Red staining

Culture plates were washed with PBS and cells were fixed in 4% formalin, followed by staining with Oil-Red-O (Sigma-Aldrich), and photographed. The dye was then extracted with 100% isopropanol and the absorbance was determined at 492 nm.

### ELISA

Serum, culture media and protein lysates samples were analyzed for adiponectin and leptin using an adiponectin mouse ELISA kit (ab108785, Abcam) and leptin mouse ELISA kit (KMC2281, Invitrogen).

### Electrophysiology

For patch clamp experiments, coverslips with cells were transferred to the recording chamber and perfused with an external Ringer's solution using a previous protocol (Sukumaran et al., 2015). Whole-cell currents were recorded using an Axopatch 200B (Axon Instruments, Inc.). The patch pipette had resistances between 3 MΩ and 5 MΩ after filling with the standard intracellular solution that contained the following: 150 mM cesium methane sulfonate, 8 mM NaCl, 10 mM HEPES, 10 mM EGTA, pH 7.2 (CsOH). Basal leak was subtracted from the final currents and average currents are shown. The maximum peak currents were calculated at a holding potential of −80 mV. The voltage–current (I–V) curves were made using a ramp protocol ranging from −100 mV to +100 mV and 100 ms duration were delivered at 2 s intervals, whereby current density was evaluated at various membrane potentials and plotted.

### PCR analysis

Total RNA was extracted using the RNeasy Lipid Tissue Mini kit and Qiacube (Qiagen) from flash-frozen subcutaneous adipose tissue. cDNA was synthesized using the Quantitect Reverse Transcriptase kit (Qiagen) Rox FastStart Universal Probe Master Mix assay reagents were purchased from Roche. Primers were purchased from Integrated DNA Technology. The endogenous control (18S rRNA) was purchased from Applied Biosystems. RT-PCR analysis for TRPC1 transcripts was done with primers from the eighth and ninth exons (Up, 5′-GCAACCTTTGCCCTCAAAGTG-3′ and Down, 5′-GGAGGAACATTCCCAGAAATTTCC-3′) after the EcoRI site (Eurofins MWG Operon).

### Statistical analysis

Mean±s.e.m. values were computed for all continuous variables, and frequency distributions were calculated for all categorical variables. Statistical comparisons were made using Student's *t*-test or one-way and two-way ANOVA. When an interaction was significant (*P*<0.05), Tukey contrasts were used to perform pairwise comparisons. All statistical tests were two-tailed with significant reported as follows: *P<0.05; **P<0.01; ***P<0.001; ****P<0.0001.

## Supplementary Material

Supplementary information
