# Supplementary material for: Ca2+ entry via TRPC1 is essential for cellular differentiation and modulates secretion via the SNARE complex
Source: J Cell Sci. 2019 Jul 1;132(13):jcs231878. doi: 10.1242/jcs.231878 (PMC6633397; doi:10.1242/jcs.231878)
Supplement: Supplementary information [file joces-132-231878-s1.pdf]

Table S1. Antibody details.

Antibody	Supplier	Catolog	Dilution (v/v)
Actin Cell	Signaling	4970S	1:1000
Adiponectin	Abcam	ab85827	1:1000
FABP4	Cell Signaling	3544T	1:1000
Leptin	Abcam	ab9749	1:1000
ORAI1	Alomone Labs	Acc-060	1:1000
ORAI3	Alomone Labs	ACC-065	1:1000
Perlipin	Cell Signaling	9349T	1:1000
PPARγ	Cell Signaling	2435T	1:1000
SNAP25	Syntaptic Systems	111-002	1:10000
STIM1	Cell Signaling	4916s	1:1000
Syntaxin1	Syntaptic Systems	110-101	1:10000
TRPC1	Alomone Labs	ACC-010	1:500
TRPC3	Alomone Labs	ACC-016	1:200
TRPC5	Alomone Labs	ACC-020	1:200
VAMP2	Syntaptic Systems	104 202	1:10000
